# Hypermobile Ehlers–Danlos Syndrome With Prominent Gastrointestinal and Autonomic Involvement in a Latin American Patient: A Case Report

**DOI:** 10.7759/cureus.93187

**Published:** 2025-09-25

**Authors:** Solón Chavarría-Aguilar, Esteban Zavaleta-Monestel, Adriana Anchía-Alfaro

**Affiliations:** 1 Internal Medicine, Hospital Clínica Bíblica, San José, CRI; 2 Pharmacy, Hospital Clínica Bíblica, San José, CRI; 3 Research, Hospital Clínica Bíblica, San José, CRI

**Keywords:** connective tissue disorders, dysautonomia, ehlers-danlos syndrome, joint instability, latin america

## Abstract

Hypermobile Ehlers-Danlos syndrome (hEDS) is a common connective tissue disorder with multisystem involvement. In Latin America, late diagnosis and limited access to specialists complicate clinical management. We present the case of a 32-year-old woman clinically diagnosed with hEDS after more than 15 years of progressive symptoms. In addition to generalized joint hypermobility (Beighton 9/9) and chronic musculoskeletal pain, she exhibited significant gastrointestinal manifestations, including refractory constipation since childhood, episodes of diarrhea, multiple food intolerances, small intestinal bacterial overgrowth (SIBO) documented twice, and suspected delayed gastric emptying. Multiple fragmented medical evaluations, combined with the patient’s persistence in seeking explanations and undergoing functional tests, ultimately allowed for a comprehensive diagnosis. Different targeted medications, stabilizing physiotherapy, an individualized exclusion diet, and nutritional support were implemented, achieving only partial improvement of symptoms.

This case illustrates how dysautonomia, connective tissue dysfunction, and gastrointestinal and metabolic alterations interact in hEDS. The coexistence of nocturnal hypoglycemia deserves particular attention, given its potential cardiovascular impact. A comprehensive interpretation of electrocardiogram (ECG), tilt-test, SIBO, and glucose monitoring provides a more complete diagnostic approach. In patients with hEDS, a multidisciplinary strategy is essential, including a metabolic assessment. Clinical and public awareness, as well as improved diagnostic access in Latin America, remain critical priorities.

## Introduction

Ehlers-Danlos syndromes (EDS) comprise a clinically and genetically heterogeneous group of hereditary connective tissue disorders. These conditions share common manifestations such as joint hypermobility, skin hyperextensibility, tissue fragility, abnormal scarring, and a tendency to develop bruising. Since connective tissue is a fundamental structural component in multiple organs and systems, clinical manifestations may affect virtually any organ or physiological function, varying in severity and presentation [1,2].

The diagnosis of EDS is based on clinical criteria, supported by family history and, in some subtypes, by genetic studies using next-generation sequencing (NGS), considered the gold standard for molecular classification [3,4]. However, the hypermobile subtype (hEDS) represents a significant exception, as it currently lacks a specific genetic biomarker, making it a predominantly clinical entity with a high risk of underdiagnosis [5].

To date, no curative treatments exist for the different subtypes of EDS; therefore, management relies on a multidisciplinary approach focused on monitoring complications, symptomatic control, and functional rehabilitation. Chronic musculoskeletal pain, dysautonomia, neuropsychiatric and gastrointestinal manifestations, as well as psychosocial impact, represent therapeutic challenges that require specialized and sustained attention [1].

Chronic pain is a frequent and disabling manifestation in EDS, whose pathophysiological mechanism remains poorly understood. Proposed treatments for different types of musculoskeletal pain have shown variable results, underscoring the urgent need to define, standardize, and evaluate current management modalities [1,6].

This case report aims to highlight the clinical complexity of hEDS, particularly in the Latin American context, and to reflect on the diagnostic, therapeutic, and structural limitations faced by patients with underdiagnosed connective tissue disorders.

## Case presentation

We present the case of a 32-year-old female patient with a clinical diagnosis of EDS, with high suspicion of hEDS. Since childhood, the patient had musculoskeletal manifestations consistent with generalized joint hypermobility. At the age of five, she required orthopedic footwear and, since the age of 11, has experienced chronic musculoskeletal pain, predominantly in the cervical and lumbar regions, accompanied by muscle fibrosis and postural instability attributed to imbalance in the activation of specific muscle groups. Table 1 summarizes the main clinical manifestations of our patient documented throughout the course of the disease.

**Table 1:** Multisystemic clinical manifestations in a patient with suspected hEDS SIBO: small intestinal bacterial overgrowth; hEDS: hypermobile Ehlers-Danlos syndrome

System	Relevant Clinical Manifestations
Musculoskeletal	Joint hypermobility (Beighton 9/9), left foot subluxation, right suprapatellar bursa effusion, right forearm fracture, left acetabular labrum tear
Skin and connective tissue	Atrophic “cigarette paper” scars, frequent bruising, bluish sclerae, dry fragile reactive skin, atopic dermatitis
Cardiovascular/Vascular	Syncope, palpitations, dizziness
Neurological/Sensory	Migraines, chronic fatigue, “brain fog,” autism level 1, episodes of anxiety/mania, sensory hypersensitivity
Gastrointestinal	Severe constipation, episodic diarrhea, positive SIBO breath test, slow gastric emptying, food intolerances, reactive hypoglycemia
Genitourinary	Stress urinary incontinence, recurrent urinary tract infections

These clinical manifestations were evaluated at different times by multiple specialists in a fragmented manner, and the underlying disorder was not recognized until recent years. Most symptoms had been present intermittently since early childhood, typically emerging in crises rather than as continuous complaints. Their severity and expression varied considerably over time, with changing combinations of musculoskeletal, gastrointestinal, and autonomic manifestations that appeared and disappeared throughout adolescence and adulthood.

**Figure 1:**
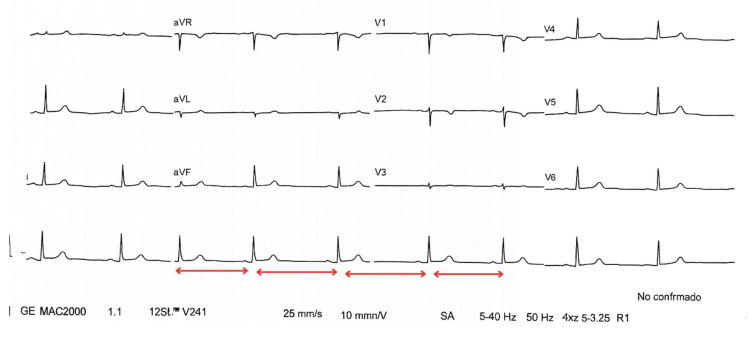
Electrocardiogram in sinus rhythm with bradycardia, the arrows illustrate reduced frequency between QRS complexes

An electrocardiogram showed sinus bradycardia (Figure 1), and a tilt-table test was performed. The test was terminated at 10 minutes of phase I due to a vasodepressor response, with a significant drop in blood pressure compared to the immediately preceding measurement. No cardioinhibitory response was observed. During the test, the patient experienced her usual symptoms-dizziness, weakness, pallor, nausea, and presyncope-confirming vasodepressor-type neurocardiogenic syncope.

In the psychiatric domain, the patient was evaluated multiple times for recurrent affective and anxiety symptoms. Early clinical assessments described episodes interpreted as mania and depression, which led to psychiatric diagnoses including bipolar disorder. These manifestations were later reconsidered, as EDS is not associated with true manic episodes. It is therefore likely that her symptoms were misunderstood and misclassified as bipolar disorder. Subsequent neuropsychological testing suggested that the patient tends to experience heightened anxiety when strong emotions threaten to override rational control, leading to anxiety crises characterized by overthinking and obsessive thoughts. A specialized evaluation ultimately established the diagnosis of autism spectrum disorder (level 1), a condition that has reported clinical associations with hEDS [7].

Due to persistent multisystem symptoms and after extensive personal research, the patient consulted an Internal Medicine specialist, who confirmed the suspicion of EDS, establishing a clinical diagnosis compatible with hEDS.

Her family history revealed multiple features suggestive of connective tissue disorders, including recurrent hernias and distinctive physical characteristics, as well as cardiovascular conditions such as heart murmurs and sudden infant death, and neuropsychiatric conditions, including attention-deficit/hyperactivity disorder (ADHD). These findings reinforce the suspicion of an underlying genetic basis. Notably, none of her relatives had ever been formally diagnosed with EDS or any other connective tissue disorder.

Among the most persistent manifestations were gastrointestinal symptoms. Since childhood, she had severe constipation associated with painful cramps and anal fissures, requiring daily laxatives between the ages of 11 and 15. During this period, she also developed skin reactions to certain foods (chocolate, strawberries, yellow dye #5), diagnosed as atopic dermatitis. As part of treatment, the physician indicated exclusion of these foods, which significantly improved her symptoms. By age 15, she was able to progressively reintroduce all food groups without new allergic exacerbations.

Currently, she continues to have significant gastrointestinal symptoms, including food aversion, constipation, constant nausea, and multiple intolerances (gluten, lactose, legumes), leading to various dietary interventions, including the low-FODMAP (Fermentable Oligo-, Di-, Mono-saccharides And Polyols) diet. However, these measures provided only temporary relief.

**Figure 2:**
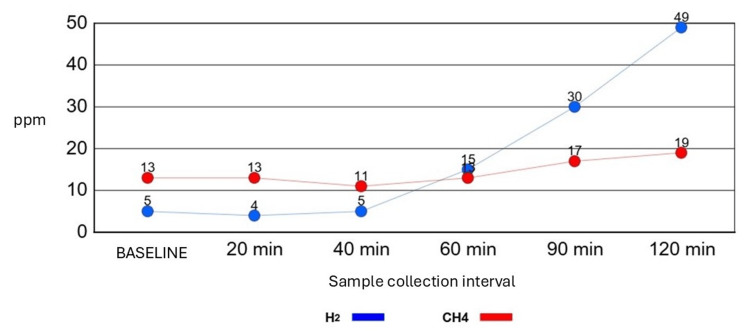
Positive small intestinal bacterial overgrowth (SIBO) breath test

**Figure 3:**
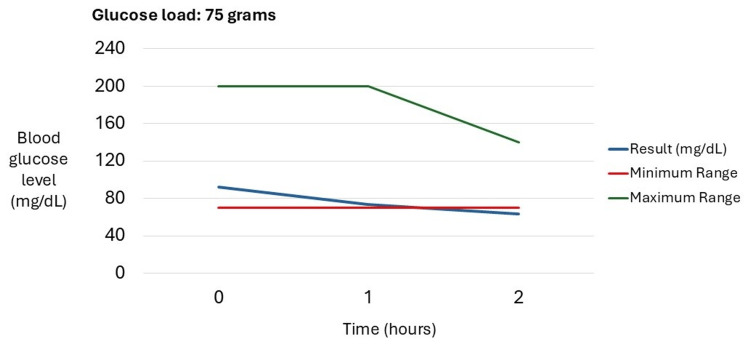
Oral glucose tolerance test (OGTT) in an adult patient after a 75 g oral glucose load, showing measured glucose levels compared with minimum and maximum reference ranges.

Recent studies confirmed small intestinal bacterial overgrowth (SIBO) with two positive breath tests performed in June and October 2024. After the first positive test, the patient completed treatment with ciprofloxacin, rifaximin, and probiotics, without clinical resolution to date (Figure 2). Gastric emptying scintigraphy is pending due to suspected gastroparesis. An oral glucose tolerance test (OGTT) with a 75 g glucose load revealed blood glucose levels below the minimum reference range at 2 hours, suggesting impaired glucose processing; further studies are planned to evaluate insulin levels and pancreatic function to rule out other comorbidities (Figure 3). In addition, predominantly nocturnal hypoglycemia was documented by the patient using a continuous glucose monitor. Hypoglycemia was defined as blood glucose levels below 70 mg/dL, accompanied by symptomatic episodes such as profuse sweating and dizziness, detected when the monitor alarm was triggered.

Due to the multisystemic nature of her clinical condition, the patient receives multidisciplinary follow-up. She is currently undergoing genetic evaluation with massive parallel sequencing to confirm the specific subtype of EDS. Her management includes weekly physiotherapy focused on musculoskeletal rehabilitation and pain control, as well as specialized nutritional support for managing food intolerances and digestive symptoms.

The functional impact has been considerable: she lost her job in 2024 due to physical limitations, has minimal physical activity and persistent fatigue, and restrictive diets have significantly affected her quality of life and psychosocial well-being. These findings emphasize the need for a comprehensive, coordinated, multidisciplinary diagnostic and therapeutic approach in patients with suspected hEDS.

## Discussion

The hypermobile subtype of EDS is the most prevalent and is characterized by wide clinical variability and multisystemic complexity, which hinder timely diagnosis and complicate comprehensive therapeutic management, making it a particularly challenging disorder [5].

In this case, the patient showed classical hEDS symptoms, including generalized joint hypermobility, maximum Beighton score (9/9), subluxations, chronic musculoskeletal pain, and autonomic dysfunction. Gastrointestinal involvement was significant, not only because of its high frequency in this entity but also because of its impact on quality of life.

Functional studies provided objective evidence of multisystemic involvement. The electrocardiogram showed sinus bradycardia at 55 beats per minute, consistent with autonomic dysfunction [8]. The tilt-table test was positive for neurocardiogenic syncope. A decade later, the patient’s medical records reported a diagnosis of postural tachycardia syndrome (POTS) by her cardiologist. However, a retrospective review of the tilt-test results and her clinical history, together with the patient’s own investigations, demonstrated that the diagnostic criteria, defined as an increase in heart rate ≥30 bpm or a maximum heart rate ≥120 bpm within 10 minutes of assuming upright posture, were not fulfilled [9]. This discrepancy highlights the inherent challenges in evaluating autonomic dysfunction in EDS, where overlapping and complex findings may lead to physician uncertainty and, in turn, to patient confusion from conflicting or inaccurate diagnoses.

Several studies have documented a high prevalence of gastrointestinal symptoms in patients with hEDS, including esophageal dysmotility, gastroparesis, severe constipation, irritable bowel syndrome, and small intestinal bacterial overgrowth [10]. In this case, the patient presented with refractory constipation since childhood, prolonged laxative use, hemorrhoids, anal fissures, episodic diarrhea, nausea, early satiety, and suspected slow gastric emptying, as well as food intolerance to gluten, lactose, and legumes. The coexistence of upper and lower gastrointestinal symptoms, along with food intolerances and intestinal dysbiosis, suggests global alteration of the gut-enteric nervous system axis, described in patients with connective tissue dysfunction [11].

Gastrointestinal involvement was supported by two positive SIBO breath tests, reflecting intestinal dysmotility secondary to connective tissue laxity and dysautonomia. This finding is consistent with reports of up to 49% prevalence of SIBO in EDS patients, particularly those with associated dysautonomic manifestations [12]. Intestinal dysmotility favors luminal stasis and microbiota alterations, causing distension, abdominal pain, and systemic symptoms. These symptoms often respond only partially to cyclic antibiotics, targeted probiotics, and dietary interventions such as a low-FODMAP diet, as seen in this case, where the patient received ciprofloxacin, rifaximin, and probiotics without clinical resolution [13,14].

Structural alterations of connective tissue can directly affect gastrointestinal integrity and function, favoring weakness of the intestinal wall, increased epithelial permeability, and visceral changes that predispose to rectal prolapse, hernias, or even intussusception [10,14,15]. Although these manifestations were not observed in this patient, her symptoms suggest advanced functional compromise of the digestive tract.

Dietary restrictions from food intolerances, combined with persistent symptoms, increase the risk of nutritional deficiencies, weight loss, and significant deterioration in quality of life. This situation is worsened in contexts with limited access to gastroenterology or nutrition specialists experienced in multisystem diseases. In this case, partial clinical improvement was achieved after multiple evaluations and implementation of an exclusion diet [16,17].

A particularly relevant finding in this patient was nocturnal reactive hypoglycemia, documented by continuous glucose monitoring, using the diagnostic threshold of blood glucose <70 mg/dL accompanied by symptoms such as profuse sweating and dizziness. Although hypoglycemia is not a recognized manifestation of EDS, several EDS-related mechanisms may contribute, including gastrointestinal dysmotility and autonomic dysfunction. Additional factors such as rapid gastric emptying (currently under evaluation), disordered eating, malabsorption, or comorbid gastrointestinal conditions could further predispose to postprandial or fasting hypoglycemia. Autonomic dysregulation and small fiber neuropathy may also blunt symptom awareness, delaying recognition of low glucose levels. The OGTT, performed as part of the broader workup, showed a blood glucose value below the minimum reference range at 2 hours post-load, suggesting impaired glucose handling. Given the potential severity of recurrent hypoglycemia, the patient is undergoing further endocrine and pancreatic evaluation to rule out alternative causes. Nonetheless, it remains possible that features of EDS may play a contributory role in this manifestation [8,18,19].

Gastrointestinal involvement in hEDS is often underestimated by clinicians, partly because of the absence of structural findings in conventional imaging studies. This underlines the importance of integrating functional tools, such as nuclear medicine gastric emptying studies, esophageal manometry, or breath tests for SIBO, into the systematic evaluation of these patients [10].

Importantly, this case highlights systemic challenges in the Latin American context, where access to specialized autonomic and gastrointestinal functional testing is limited, and patients frequently experience fragmented care across multiple providers. These structural and healthcare barriers contribute to significant diagnostic delays, under-recognition of multisystemic involvement, and difficulties in implementing coordinated, multidisciplinary management strategies. Documenting such cases emphasizes the need to improve awareness, access to diagnostic resources, and continuity of care for patients with hEDS in resource-limited settings.

## Conclusions

This case underscores the need for early recognition and multidisciplinary management in hypermobile EDS, particularly in resource-limited settings where diagnostic and therapeutic options are constrained. Comprehensive evaluation should extend beyond musculoskeletal and gastrointestinal systems to include autonomic assessments, given the frequent overlap of dysautonomia and related complications. Proactive gastrointestinal evaluation and coordinated care involving gastroenterology, genetics, physiatry, psychology, and nutrition are key to improving outcomes. As most management recommendations currently rely on expert opinion, observational data, or small studies, there remains a critical need for further research. To optimize care, strengthening medical education, expanding access to specialized testing, and implementing standardized protocols are essential to ensure equitable and comprehensive management of connective tissue disorders.
